# Speaker demographics at the Society for Healthcare Epidemiology of America (SHEA) Spring Conference, 2019–2022

**DOI:** 10.1017/ash.2023.347

**Published:** 2023-09-29

**Authors:** Catherine J. Cichon, Elizabeth R. Lyden, Ibukunoluwa C. Kalu, Jonathan Herskovitz, Jacinda C. Abdul-Mutakabbir, Zanthia Wiley, Jasmine R. Marcelin

## Abstract

**Background:** The Society for Healthcare Epidemiology of America (SHEA) serves as a national platform for infection prevention and antibiotic stewardship. Like many professional healthcare societies over the last decade, the SHEA has pledged to provide equitable opportunities to individuals in the organization. The impact of these efforts remains undetermined. This study evaluated trends in speaker demographics at the annual SHEA Spring Conference from 2019 to 2022. **Methods:** SHEA leadership or staff provided demographic information on SHEA members and Spring conference speakers (excluding poster sessions) from 2019 to 2022. We excluded 2020 due to conference cancellation. Data were summarized using descriptive statistics, and χ^2^ analysis was used to evaluate changes over time. Individual speakers were compared with member demographics. Self-reported SHEA speaker and member demographics were available for sex, race or ethnicity, age, primary practice setting, and professional degrees. Speaker professional degree was not available for 2022. **Results:** In total, 447 speaker slots were filled by 218 unique speakers over the 3-year period. The SHEA average annual membership between 2019 and 2022 with self-reported demographics included 55.2% female and 44.8% male members, with race reported as follows: 69.3% White, 21.4% Asian, 6.0% Hispanic or Latino, 2.9% Black, 0.4% American Indian/Alaska Native/Native Hawaiian/Pacific Islander (AIAN/NHPI). However, almost half of the members did not report a race or ethnicity. The SHEA speakers during the same period were 63.5% female and 36.5% male, with 68.2% White, 13.3% Asian, 3.8% Black, 3.4% Hispanic/Latino, and 0.8% AIAN/NHPI. Only 13.4% of speakers did not report race or ethnicity. Every year, there were fewer than 6 speakers in each of the Black, Hispanic or Latino, AIAN/NHPI race or ethnicity categories. In 2019, 49.2% of speakers were aged 41–50 years, compared with 28.6% of members in that age group (*P* = 0.0029). By 2022, 35.6% of speakers were aged 41–50 years, compared with 29.3% of members in that age group (*P* = .074). In 2021, pharmacists represented 11.9% of speakers compared with 2.9% of members, and members with nondoctoral degrees represented 11.1% of speakers compared with 21.4% of members (*P* < .0001). In each year, there was a statistically significant association between primary practice setting and speaker or member representation, with underrepresentation of community or private-practice speakers relative to their proportion of membership: 2019 (7.5% speakers vs 14.3% members), 2021 (6.5% speakers vs 15.2% members), 2022 (4.3% speakers vs 15.7% members) (*P* < .05) . **Conclusions:** Although there has been more equitable speaker age representation and an increase in pharmacist speakers at the SHEA Spring Conference over time, practitioners from community settings and those with nondoctoral degrees remain underrepresented relative to the SHEA membership. Racial or ethnic minoritized individuals remain underrepresented as members and speakers compared with the general US population. Intentional interventions are needed to consistently achieve equitable speaker representation across multiple demographic groups at the SHEA Spring Conference.

**Disclosures:** None

